# Revaccination with Bacille Calmette-Guérin (BCG) is associated with an increased risk of abscess and lymphadenopathy

**DOI:** 10.1038/s41541-021-00421-5

**Published:** 2022-01-14

**Authors:** Paola Villanueva, Ushma Wadia, Nigel Crawford, Nicole L. Messina, Tobias R. Kollmann, Michaela Lucas, Laurens Manning, Peter Richmond, Laure F. Pittet, Nigel Curtis

**Affiliations:** 1 Department of Paediatrics, The University of Melbourne, Parkville, VIC Australia; 2 Infection and Immunity, Murdoch Children’s Research Institute, Parkville, VIC Australia; 3 Department of General Medicine, Royal Children’s Hospital, Melbourne, Parkville, VIC Australia; 4 Wesfarmers Centre for Vaccines and Infectious Diseases, Telethon Kids Institute, Perth, WA Australia; 5 Immunisation Service, Royal Children’s Hospital Melbourne, Parkville, VIC Australia; 6 School of Medicine, University of Western Australia, Perth, WA Australia; 7 Department of Immunology, Sir Charles Gairdner Hospital, Perth, WA Australia; 8 Departments of Immunology and General Paediatrics, Perth Children’s Hospital, Perth, WA Australia; 9 Department of Immunology, Pathwest, QE2 Medical Centre, Perth, WA Australia; 10 Department of Infectious Diseases, Fiona Stanley Hospital, Perth, WA Australia; 11 Infectious Diseases, Royal Children’s Hospital Melbourne, Parkville, VIC Australia

**Keywords:** Outcomes research, Live attenuated vaccines

## Abstract

The reported frequency and types of adverse events following initial vaccination and revaccination with Bacille Calmette-Guérin (BCG) varies worldwide. Using active surveillance in a randomised controlled trial of BCG vaccination (the BRACE trial), we determined the incidence and risk factors for the development of BCG injection site abscess and regional lymphadenopathy. Injection site abscess occurred in 3% of 1387 BCG-vaccinated participants; the majority (34/41, 83%) resolved without treatment. The rate was higher in BCG-revaccinated participants (OR 3.6, 95% CI 1.7–7.5), in whom abscess onset was also earlier (median 16 vs. 27 days, *p* = 0.008). No participant with an abscess had a positive interferon-gamma release assay. Regional lymphadenopathy occurred in 48/1387 (3%) of BCG-vaccinated participants, with a higher rate in revaccinated participants (OR 2.1, 95% CI 1.1–3.9). BCG-associated lymphadenopathy, but not injection site abscess, was influenced by age and sex. A previous positive tuberculin skin test was not associated with local reactions. The increased risk of injection site abscess or lymphadenopathy following BCG revaccination is relevant to BCG vaccination policy in an era when BCG is increasingly being considered for novel applications.

## Introduction

The most common adverse reactions to Bacille Calmette-Guérin (BCG) vaccination are injection site abscess and regional lymphadenitis^1,2^. The reported frequency of these local adverse events following immunisation (AEFI) varies worldwide, likely attributable to different surveillance methods, case definitions, vaccine dose and strain, vaccine administration route, as well as host immune status^3–5^.

Using standard case definitions and active surveillance data from a multicentre randomised controlled trial of BCG vaccination to reduce the impact of COVID-19 in healthcare workers (the BRACE trial; ClinicalTrials.gov NCT04327206; date of registration 31 March 2020), we aimed to determine the incidence, and the risk factors for the development, of BCG injection site abscess and regional lymphadenopathy.

## Results

**Fig. 1:**
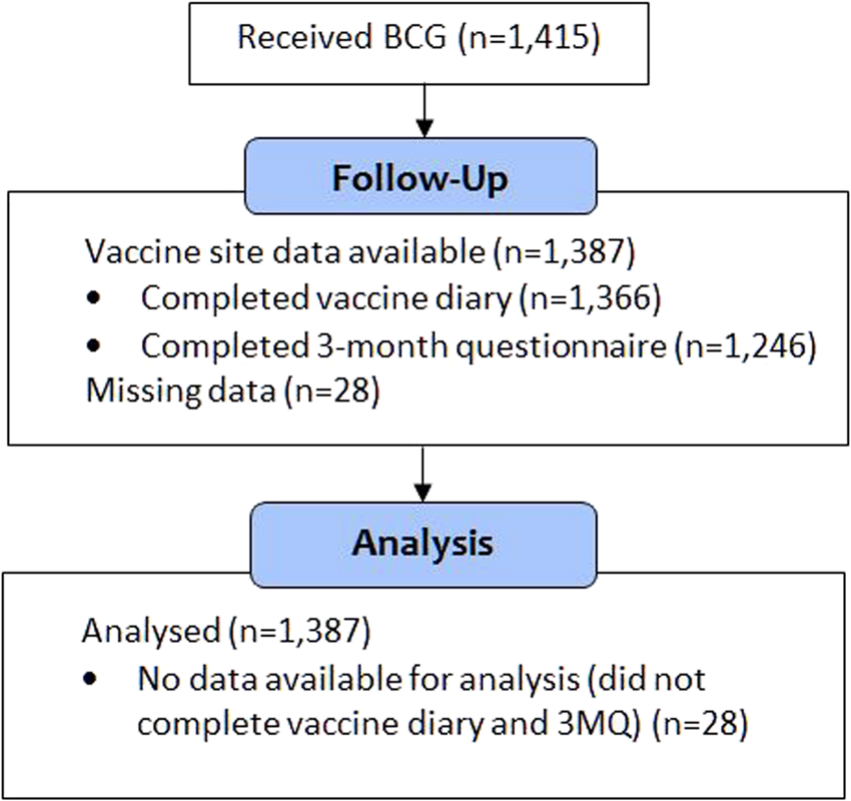
BRACE stage 1 participants who received BCG. BCG Bacille Calmette-Guérin, 3MQ 3-month questionnaire.

Among the 1415 participants who received BCG, 1387 (98%) provided vaccine safety data (Fig. 1). Vaccinees ranged in age from 19 to 74 years old (median 41) and comprised predominantly healthcare workers (HCW) in clinical roles (77% nurses, doctors or allied health clinicians) (Table 1). The majority were female (76%) and a half (51%) had a prior history of BCG vaccination.

**Table 1:** Factors investigated for association with the development of BCG adverse reaction.

Factor	Total	BCG injection site abscess				BCG-associated lymphadenopathy			
	BCG		Univariate	Univariate	Multivariate		Univariate	Univariate	Multivariate
	*n* = 1387	*n* = 41 (%)	OR (95% CI)	Adjusted^a^ OR (95% CI)	OR (95% CI)	*n* = 48 (%)	OR (95% CI)	Adjusted^a^ OR (95% CI)	OR (95% CI)
Sex									
Female	1048	32 (3.0)	1 (reference)	1 (reference)	—	43 (4.1)	1 (reference)	1 (reference)	1 (reference)
Male	339	9 (2.7)	0.87 (0.41–1.83), *p* = 0.71	0.83 (0.39–1.76), *p* = 0.62		5 (1.5)	0.35 (0.14–0.89), *p* = 0.03	0.34 (0.13–0.87), *p* = 0.02	0.33 (0.13–0.86), *p* = 0.02
Age									
18–49	932	29 (3.1)	1 (reference)	1 (reference)	—	39 (4.2)	1 (reference)	1 (reference)	1 (reference)
50+	455	12 (2.6)	0.84 (0.43–1.67), *p* = 0.63	0.57 (0.28–1.16), *p* = 0.12		9 (2.0)	0.46 (0.22–0.95), *p* = 0.04	0.35 (0.17–0.78), *p* = 0.007	0.35 (0.16–0.75), *p* = 0.007
State									
Western Australia	891	16 (1.8)	1 (reference)	1 (reference)	—	36 (4)	1 (reference)	1 (reference)	—
Victoria	496	25 (5.0)	2.91 (1.54–5.51), *p* = 0.001	3.20 (1.68–6.09), *p* < 0.001		12 (2.4)	0.59 (0.30–1.14), *p* = 0.12	0.61 (0.31–1.19), *p* = 0.15	
Study site									
A	268	9 (3.4)	1.18 (0.56–2.50), *p* = 0.67	1.15 (0.54–2.45), *p* = 0.72	1.15 (0.54–2.45), *p* = 0.72	13 (4.9)	1.58 (0.82–3.03), *p* = 0.17	1.56 (0.81–2.99), *p* = 0.18	—
B	362	2 (0.6)	0.14 (0.03–0.58), *p* = 0.007	0.13 (0.03–0.55), *p* = 0.006	0.13 (0.03–0.55), *p* = 0.006	16 (4.4)	1.43 (0.78–2.65), *p* = 0.25	1.41 (0.76–2.60), *p* = 0.28	
C	261	5 (1.9)	0.59 (0.23–1.52), *p* = 0.28	0.57 (0.22–1.48), *p* = 0.25	0.57 (0.22–1.48), *p* = 0.25	7 (2.7)	0.73 (0.32–1.64), *p* = 0.45	0.72 (0.32–1.62), *p* = 0.43	
D	368	22 (6.0)	3.35 (1.79–6.26), *p* < 0.001	3.94 (2.09–7.44), *p* < 0.001	3.94 (2.09–7.44), *p* < 0.001	10 (2.7)	0.72 (0.36–1.46), *p* = 0.37	0.76 (0.37–1.55), *p* = 0.45	
E	113	3 (2.7)	0.89 (0.27–2.92), *p* = 0.84	0.80 (0.24–2.65), *p* = 0.72	0.80 (0.24–2.65), *p* = 0.72	1 (0.9)	0.23 (0.03–1.71), *p* = 0.15	0.22 (0.03–1.62), *p* = 0.14	
F	15	0 (0.0)	—	—	—	1 (6.7)	2.01 (0.26–15.63), *p* = 0.50	2.06 (0.26–16.08), *p* = 0.49	
Role									
Nurse/midwife	569	17 (3.0)	1.02 (0.54–1.91), *p* = 0.95	1.08 (0.57–2.03), *p* = 0.82	—	22 (3.9)	1.23 (0.69–2.18), *p* = 0.49	1.26 (0.70–2.24), *p* = 0.44	1.01 (0.56–1.83), *p* = 0.98
Medical practitioner	261	13 (5.0)	2.06 (1.05–4.03), *p* = 0.04	1.81 (0.92–3.56), *p* = 0.09		11 (4.2)	1.30 (0.65–2.57), *p* = 0.46	1.22 (0.61–2.43), *p* = 0.58	1.48 (0.72–3.05), *p* = 0.28
Allied health	230	4 (1.7)	0.54 (0.19–1.51), *p* = 0.24	0.55 (0.19–1.56), *p* = 0.26		4 (1.7)	0.45 (0.16–1.26), *p* = 0.13	0.45 (0.16–1.27), *p* = 0.13	0.42 (0.15–1.20), *p* = 0.11
Administrative/clerical	190	4 (2.1)	0.67 (0.24–1.91), *p* = 0.46	0.69 (0.24–1.97), *p* = 0.49		5 (2.6)	0.73 (0.28–1.85), *p* = 0.50	0.73 (0.29–1.88), *p* = 0.52	0.87 (0.34–2.26), *p* = 0.78
Scientist (medical/research)	45	1 (2.2)	0.74 (0.10–5.50), *p* = 0.77	0.65 (0.09–4.86), *p* = 0.68		6 (13.3)	4.76 (1.91–11.86), *p* = 0.001	4.53 (1.81–11.32), *p* = 0.001	4.30 (1.70–10.88), *p* = 0.002
PSA/hospital maintenance	78	1 (1.3)	0.41 (0.06–3.04), *p* = 0.38	0.45 (0.06–3.36), *p* = 0.44		0 (0.0)	—	—	—
Dentist/dental therapy	6	1 (16.7)	6.71 (0.77–58.72), *p* = 0.09	5.96 (0.66–53.77), *p* = 0.11		0 (0.0)	—	—	—
Other	8	0 (0.0)	—	—		0 (0.0)	—	—	—
BCG history									
1st BCG	673	10 (1.5)	1 (reference)	1 (reference)	1 (reference)	18 (2.7)	1 (reference)	1 (reference)	1 (reference)
BCG revaccination	714	31 (4.3)	3.01 (1.46–6.19), *p* = 0.003	3.01 (1.46–6.19), *p* = 0.003	3.61 (1.74–7.51), *p* = 0.001	30 (4.2)	1.60 (0.88–2.89), *p* = 0.12	1.60 (0.88–2.89), *p* = 0.12	2.11 (1.14–3.95), *p* = 0.02
Vaccinator experience									
Experienced	563	25 (4.4)	1 (reference)	1 (reference)	—	14 (2.5)	1 (reference)	1 (reference)	—
Trained	743	14 (1.9)	0.41 (0.21–0.80), *p* = 0.009	0.39 (0.13–2.37), *p* = 0.006		31 (4.2)	1.71 (0.90–3.24), *p* = 0.10	1.68 (0.88–3.18), *p* = 0.12	
Learner	81	2 (2.5)	0.54 (0.13–2.34), *p* = 0.42	0.55 (0.13–2.37), *p* = 0.42		3 (3.7)	1.51 (0.42–5.37), *p* = 0.53	1.52 (0.43–5.40), *p* = 0.52	
BCG batch									
118006D Exp 08/2020	675	27 (4.0)	1 (reference)	1 (reference)	—	22 (3.3)	1 (reference)	1 (reference)	—
118017 F Exp 11/2020	683	14 (2.0)	0.50 (0.26–0.97), *p* = 0.04	0.46 (0.24–0.89), *p* = 0.02		24 (3.5)	1.08 (0.60–1.95), *p* = 0.80	1.04 (0.58–1.88), *p* = 0.89	
118017H Exp 11/2020	29	0 (0.0)	—	—		2 (6.9)	2.20–0.49–9.83), *p* = 0.30	2.06 (0.46–9.26), *p* = 0.35	
Satisfactory vaccination									
Yes	1205	32 (2.7)	1 (reference)	1 (reference)	—	44 (3.7)	1 (reference)	1 (reference)	—
No	22	1 (4.5)	1.75 (0.23–13.38), *p* = 0.59	1.81 (0.23–14.09), *p* = 0.57		1 (4.5)	1.26 (0.17–9.55), *p* = 0.83	1.27 (0.17–9.71), *p* = 0.82	
Unknown	160	8 (5.0)	1.93 (0.87–4.27), *p* = 0.10	2.29 (1.02–5.11), *p* = 0.04		3 (1.9)	0.50 (0.15–1.64), *p* = 0.26	0.54 (0.16–1.76), *p* = 0.3	
Lived in TB endemic country									
No	1189	34 (2.8)	1 (reference)	1 (reference)	—	43 (3.6)	1 (reference)	1 (reference)	—
Yes	171	7 (4.0)	1.45 (0.63–3.32), *p* = 0.38	1.12 (0.48–2.60) *p* = 0.80		5 (2.9)	0.80 (0.31–2.04), *p* = 0.64	0.69 (0.27–1.80), *p* = 0.45	
Unknown	27	0 (0.0)	—	—		0 (0.0)	—	—	
Previous known LTBI									
No	1357	41 (3.0)	—	—	—	46 (3.4)	1 (reference)	1 (reference)	—
Yes	18	0 (0.0)				2 (11.1)	3.56 (0.80–15.95), *p* = 0.10	3.26 (0.72–14.71), *p* = 0.12	
Unknown	12	0 (0.0)				0 (0.0)	—	—	
Previous TST									
Negative/None	1032	38 (3.7)	1 (reference)	1 (reference)	—	34 (3.3)	1 (reference)	1 (reference)	—
Positive (>5mm)	103	3 (2.9)	0.78 (0.24–2.59), *p* = 0.69	0.52 (0.16–1.75), *p* = 0.29		4 (3.9)	1.19 (0.41–3.41), *p* = 0.75	1.04 (0.49–2.19), *p* = 0.93	
Unknown	252	0 (0.0)	—	—		10 (4.0)	1.21 (0.59–2.49), *p* = 0.60	—	

*BCG* Bacille Calmette-Guérin, *LTBI* latent tuberculosis infection, *OR* odds ratio, *PSA* patient services assistant, *TB* tuberculosis, *TST* tuberculin skin test.

^a^Adjusted for revaccination.

### BCG injection site abscess

**Fig. 2:**
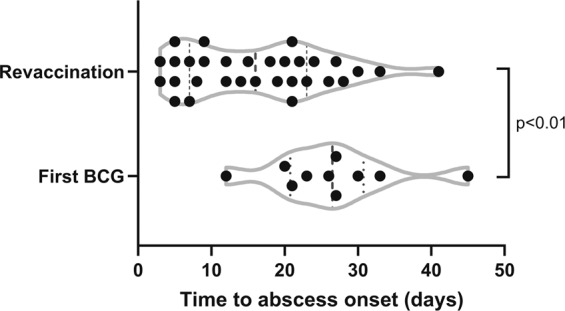
Injection site abscess onset. Time at which injection site abscess apparent after BCG vaccination.

BCG injection site abscess occurred in 41/1387 (3.0%) participants: 10 of 673 (1.5%) BCG-naïve participants; 31 of 714 (4.3%) with a prior history of BCG vaccination (Table 1). Of the 1387 participants, 1200 (87%) reported erythema and/or swelling at the injection site, but did not meet the criteria for BCG injection site abscess. The median time to abscess onset was 20 days (interquartile range [IQR] 9–26) (Table 2), occurring earlier in revaccinated participants (median 16 days (IQR 8–23) vs. 27 days (IQR 22–30), *p* = 0.008) (Fig. 2). In BCG-revaccinated participants, the previous dose had been administered 9 to 55 years (median 34) prior. Five participants had two prior BCG vaccinations.

**Table 2:** Clinical features of BCG local adverse reactions.

Injection site abscess	Any BCG dose	First BCG	BCG revaccinated	*p* value
	*n* = 41	*n* = 10	*n* = 31	
Clinical features				
Time to onset, days	20 (3–45)	27 (12–45)	16 (3–41)	0.008
Maximum size, cm	2 (1.5–5.0)	2.5 (1.5–5.0)	2 (1.5–4.5)	0.12
Abscess with discharge	41 (100%)	10 (100%)	31 (100%)	
Abscess with persistent discharge (>2w)	24 (59%)	5 (50%)	19 (61%)	0.39
Abscess with pain/tenderness at site	40 (98%)	10 (100%)	30 (97%)	
Management				
Observation	34 (83%)	7 (70%)	27 (87%)	
Maximum size, cm	2 (1.5–5.0)	2 (1.5–5.0)	2 (1.5–4.0)	
Time to resolution, days	27 (2–243)	28 (8–81)	22 (2–243)	0.43
Antimicrobial only	5 (12%)	2 (20%)	3 (10%)	
Topical antibiotic (mupirocin)	1	0	1	
Maximum size, cm	2.5	—	2.5	
Time to resolution, days	28	—	28	
Oral antibiotics (cephalexin/flucloxacillin)	2	1	1	
Maximum size, cm	3.5	5.0	2.0	
Time to resolution, days	15 (8–21)	8	21	
Oral isoniazid	2	1	1	
Maximum size, cm	4.3 (4.0–4.5)	4.0	4.5	
Time to resolution, days	131	113	149	
Fine needle aspiration + cephalexin	2 (5%)	1 (10%)	1 (3%)	
Maximum size, cm	2.0 (2.0–2.0)	2.0	2.0	
Time to resolution, days	43 (30–56)	56	30	

Lymphadenopathy	Any BCG dose	First BCG	BCG revaccinated	*p* value
	*n* = 48	*n* = 18	*n* = 30	
Clinical features				
Time to onset, days	6 (1–56)	6 (1–56)	6 (1–42)	0.29
Maximum size, cm	1.8 (0.5–4.0)	1.8 (0.5–4.0)	1.5 (0.5–3.0)	0.46
No. (%) with pain/tenderness at site	30 (63%)	14 (78%)	16 (53%)	0.13
Time to resolution, days	4 (1–30)	6 (1–30)	3 (1–14)	0.47

Categorical variables are reported as number (%), continuous variables are reported as median (range).

**Fig. 3:**
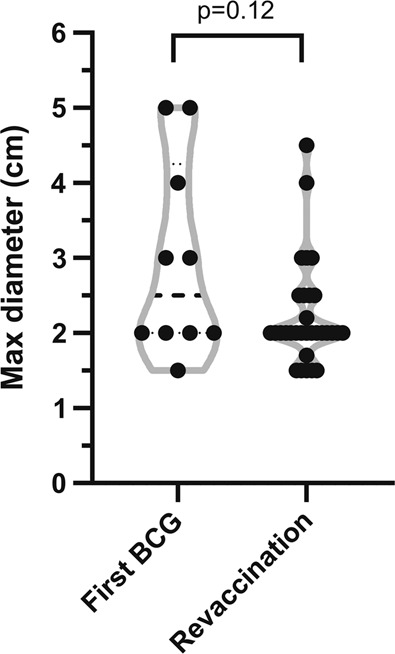
Injection site abscess size. Maximum diameter of injection site abscess.

The median diameter was 2.0 cm (IQR 2.0–2.5) (Fig. 3). All abscesses, except for one, were discharged (most commonly ‘yellow cloudy’ fluid) and 24/41 (59%) had persistent discharge for more than 2 weeks. All participants, except for one, experienced pain or tenderness at the abscess site. One participant with a 5.0 cm abscess presented to an emergency department with severe injection site pain. Three participants, with 4.0, 2.5 or 2.0 cm abscess each, had associated axillary lymphadenopathy.

The safety medical doctors (SMDs) recommended a conservative approach for all, except two participants who were referred to an Infectious Diseases specialist due to persistent large abscesses (≥4.0 cm diameter). External providers prescribed antimicrobial treatment in 7/41 (17%) participants, including two who additionally self-performed fine needle aspiration and two who received isoniazid treatment (both negative interferon-gamma release assay (IGRA) results). The remaining cases (34/41, 83%) resolved spontaneously without treatment, in a median time of 27 days (IQR 11–45).

### Factors associated with the development of BCG injection site abscess

In the univariate analysis (Table 1), an abscess was more common among medical practitioners compared to other HCW, those with a history of prior BCG vaccination, and those in study site D. An abscess was less likely in participants vaccinated by trained vaccinators compared with experienced vaccinators, with BCG batch 118017 F and in study site B. All factors remained significant when adjusted for revaccination, except for the participant role. In the multivariate analysis, revaccination (OR 3.61, 95% CI 1.74–7.51) and study site (site D, OR 3.94, 95% CI 2.09–7.44) were the only two factors associated with an injection site abscess.

There was no significant association with age group, sex, history of living in tuberculosis (TB) endemic country or prior positive tuberculin skin test (TST). All participants with an injection site abscess had no known previous latent tuberculosis infection (LTBI). Of the 27 (67%) participants who developed an injection site abscess and had an IGRA test 3 months post vaccination, all had negative IGRA results.

### BCG-associated lymphadenopathy

Lymphadenopathy was reported in 48/1387 (3%) BCG-vaccinated participants. Reported lymphadenopathy comprised: ipsilateral axillary (*n* = 22, 46%), axillary and cervical (*n* = 6, 12%), axillary and supraclavicular (*n* = 1, 2%), cervical (*n* = 15, 31%), submandibular (*n* = 2, 4%) or supraclavicular (*n* = 2, 4%). The median time to onset was 6 days (IQR 3–9) after vaccination (Table 2).

The median diameter was 1.8 cm (IQR 1–2), and 30/48 (63%) participants experienced overlying tenderness. None were suppurative or had overlying redness. All self-resolved, in a median time of 3 days (IQR 2–8). One participant was treated with isoniazid for a concomitant injection site abscess of 4.0 cm diameter.

Two participants took prescribed opioid analgesia for axillary pain associated with lymphadenopathy; one took codeine for two days and the other for buprenorphine for four days following an emergency department presentation.

### Factors associated with the development of BCG lymphadenopathy

In the univariate analysis (Table 1), lymphadenopathy was more common among laboratory and research scientists, and less likely in males and in older participants. All factors remained significant when adjusted for revaccination. In the multivariate analysis, revaccination (OR 2.11, 95% CI 1.14–3.95), in addition to scientist role (OR 4.3, 95% CI 1.70–10.88), male sex (OR 0.33, 95% CI 0.13–0.86) and older age group (OR 0.35 95% CI 0.16–0.75) influenced the risk of lymphadenopathy.

There was no significant association with study site, BCG batch, vaccinator experience, history of living in TB endemic country, prior positive TST or LTBI.

## Discussion

In this large study of over 1000 HCW recruited in Australia and actively followed for AEFI, we assessed the incidence and factors predictive of BCG injection site abscess and regional lymphadenopathy.

Studies reporting BCG injection site abscesses are scarce. However, the observed rate of injection site abscess in our study is similar to the reported 2.5% incidence in another Australian study in which half of the participants were children^1^ and 2.5% in a French study^6^ in children (Table 3).

**Table 3:** Previously reported incidence of BCG-related adverse reactions.

Source country BCG strain age at BCG^a^	BCG	Injection site abscess		Regional lymphadenopathy		
		*n*/*N*	Risk (95% CI)	*n*/*N*	Risk (95% CI)	Comments for lymphadenopathy
**Adults**						
BRACE Australia BCG-Denmark (AJ Vaccines) 19–74 y	All	41/1387	3.0% (21/1000 to 38/1000)	48/1387	3.5% (33/1000 to 36/1000)	Any size
	V	10/673	1.5% (6/1000 to 24/1000)	18/673	2.7% (14/1000 to 39/1000)	All were non-suppurative
	R	31/714	4.3% (28/1,000 to 58/1000)	30/714	4.2% (27/1,000 to 57/1000)	
Hatherill et al.^34^*,* 2014 South Africa BCG-Denmark (SSI) 18–40 y	R	NA	—	10/72	14% (53/1,000 to 225/1000)	All 72 axillary and had prior positive TST >15 mm
**All ages**						
Product Characteristics^9^ BCG-Denmark (AJ vaccines)	NA	—	<0.1% (≥0.1/1000 to <1/1000)	—	<1% (≥1/1000 to <10/1000)	Size >1.0 cm
Turnbull et al.^1^*,* 2002 Australia BCG Connaught (Montreal strain) 1d–54y	All	23/918	2.5% (15/1000 to 35/1000)	10/918	1.1% (4/1000 to 18/1000)	Size ≥1.5 cm
Ponnighaus et al.^35^*,* 1993 Malawi BCG-Glaxo 0–71 y	R	NA	—	4/54,865	0.01% (0.001/1000 to 0.14/1000)	Only included suppurative lymphadenitis
**Adolescents**						
Nemes et al.^15^*,* 2018 South Africa BCG-Denmark (SSI) 12–17 y	R	NA	—	2/330	0.6% (<1/1000 to 14/1000)	—
Dourado et al.^37^*,* 2003 Brazil BCG-Moreau 7–14 y	All V R	NA	—	7/71347 2/11,981 5/59,366	0.01% (0.03/1000 to 0.17/1000) 0.02% (<1/1000 to 0.40/1000) 0.01% (0.01/1000 to 0.16/1000)	Axillary
Cunha et al.^38^*,* 2008 Brazil BCG-Moreau 7–14 y	R	8/47,307	0.02% (0.05/1000 to 0.3/1,1000)	1/47,307	0.002% (<1/1000 to 0.06/1000)	Axillary
**Children**						
Aydinlioglu et al.^39^*,* 1993 Turkey BCG-Pasteur 3–12 m	All	NA	—	15/219	6.8% (34/1000 to 103/1000)	4 of 15 were suppurative lymphadenitis
Roth et al.^11^*,* 2010 Guinea-Bissau BCG-Denmark (SSI) 19m–5y	All V R	NA	—	38/787 14/393 24/394	4.8% (33/1000 to 64/1000) 3.6% (17/1000 to 54/1000) 6.1% (37/1000 to 85/1000)	Axillary LN >1.5 cm
Lotte et al.^5^*,* 1984 17 countries BCG intradermal, various strains <12 m	All	NA	—	3/4.8mill to 9994/263,000	Range <0.0001% (East Germany) to 3.8% (Algeria)	Only included suppurative lymphadenitis
Chaves-Carballo et al.^40^*,* 1972 Panama (BCG strain NR) Children	V^b^	NA	—	25/1295	1.9% (12/1000 to 27/1000)	Size ≥1.0 cm 12 of 25 were suppurative lymphadenitis
Kim et al.^41^*,* 2016 Korea BCG-Denmark Children	V^b^	NA	—	5/721	0.7% (<1/1000 to 13/1000)	Any size 3 of 5 were ≥1.5 cm
Nissen et al.^42^*,* 2016 Denmark BCG-Denmark (SSI)	V	NA	—	13/2118	0.6% (3/1000 to 9/1000)	Size >1.0 cm 10 of 13 were suppurative lymphadenitis
**Neonates**						
Jeena et al.^36^*,* 2001 South Africa BCG-Denmark (SSI) Neonates	V	123/9763	1.3% (10/1000 to 15/1000)	54/9763	0.6% (4/1000 to 7/1000)	Size >1.5 cm
Dommergues et al.^6^*,* 2009 France BCG-Denmark (SSI) 1d–6y	V^b^	60/2435	2.5% (18/1000 to 31/1000)	3/2435	0.1% (<1/1000 to 3/1000)	Size >1.0 cm All three were non-suppurative lymphadenitis

Abscess minimum diameter size only specified for BRACE Trial (≥1.5 cm) and Dommergues et al. (>1.0 cm).

*BCG* Bacille Calmette-Guérin, *d* days, *SPC* summary of product characteristics, *SSI* Statens Serum Institute, *LN* lymph node, *m* months, *mill* million, *n* number of cases, *N* number of vaccinees, *NA* not reported, *R* revaccinated, *TST* tuberculin skin test, *V* first BCG vaccination, *y* years.

^a^Latest dose.

^b^Presumed as children <6 years and revaccination not specified.

Despite the persistence of discharge from the injection site abscess in half of the affected participants, the majority of abscesses healed within a month without medical/surgical intervention. In those that were treated, a variety of management strategies were used, highlighting the lack of robust evidence for optimal treatment^7^. Two trial participants undertook self-directed aspiration of the abscess. No side effects of treatment were reported by these participants.

Definitions for BCG-associated lymphadenopathy vary in the literature^8^. Ipsilateral regional lymph node enlargement has also been called ‘BCG-associated regional lymphadenitis’, which can be non-suppurative or suppurative, the latter potentially causing significant morbidity. Non-suppurative lymphadenitis (or lymphadenopathy) is commonly considered a normal reaction to BCG vaccination and usually has a benign course with resolution over time, especially if infracentimetric^8,9^. However, according to the WHO, any BCG-associated local lymphadenitis is a reportable AEFI^10^.

The incidence of BCG-associated lymphadenopathy in our study was higher than has been previously reported in studies that include both adults and children (Table 3), although data from studies including exclusively adults is limited. In a study in adults with a history of latent TB infection in South Africa, a rate of 14% was reported but the study included only 72 individuals. A large review^5^ of BCG AEFI from 17 countries reported risk in infants ranging from <0.0001% (East Germany) to 3.8% (Algeria). However, these risks were solely for suppurative lymphadenitis, and were for different time periods in the 1950s to 1970s, using unspecified surveillance methods.

In our study, even though many participants experienced pain or tenderness in their regional enlarged lymph glands, none reported symptoms or signs suggestive of a suppurative lymphadenitis. The higher incidence in our study is likely to be the result of the active surveillance used, with systematic and frequent questioning of participants, a lower threshold for defining lymphadenopathy using a broader definition than previous studies and the higher dose of BCG used in adults compared to studies in infants and children.

The higher incidence of local AEFI in BCG-revaccinated participants is consistent with the higher rate of BCG-associated lymphadenopathy reported in a smaller study in children in Guinea-Bissau^11^. BCG revaccination programmes have been generally discontinued due to a lack of evidence for efficacy against TB^12,13^. However, revaccination has been recently reconsidered for the protection of adolescents^14,15^. In addition, there is growing interest in the broader applications of BCG for its beneficial ‘off-target’ effects on the immune system that protect against a variety of infections, including COVID-19^16,17^, and its role in the management of autoimmune conditions such as diabetes^18^. For these off-target effects, BCG revaccination may be required and the increased risks of injection site abscess and regional lymphadenopathy are therefore relevant.

To prevent the risk of AEFI, TST screening of adults prior to BCG vaccination or revaccination has been suggested. We did not find any association between prior history of a positive TST and local adverse event following BCG, in line with the previous studies^5^. Therefore, TST screening is unlikely to be helpful in screening subjects prior to BCG vaccination.

In addition, none of the participants who developed an injection site abscess had a positive IGRA when tested 3 months after vaccination, suggesting abscess development is unlikely to be associated with LTBI (in a low TB-prevalence setting such as Australia).

Factors associated with the development of BCG injection site abscess included the study site as well as other determinants. Participants vaccinated in one study site were more likely to develop an abscess. In a study in Denmark that found scar prevalence after BCG vaccination varied by study site, it was suggested that differences between vaccinators’ technique was an explanation^19^. In our study, the proportion of trained and experienced vaccinators varied by study site. In univariate analysis, participants vaccinated by ‘inexperienced’ vaccinators were less likely to develop an abscess than those vaccinated by ‘experienced’. This is somewhat counterintuitive as incorrect administration has been linked to injection site reactions and lymphadenitis^2^. However, this association did not persist in the multivariate analysis. It is difficult to explain this finding but it is possible that the recently trained vaccinators rapidly gained expertise during the trial.

Batch variability has been shown to affect the frequency of AEFI^20^. Each study site used two to three different batches. The higher rate of abscess formation with one particular vaccine batch in the univariate analysis was no longer present in the multivariate analysis suggesting the disproportionate use of this batch in one site explained the initial finding.

Factors associated with the development of BCG lymphadenopathy included sex and age. Our finding that lymphadenopathy was more common in females than males is consistent with previous reports of more frequent reporting of AEFI by females^1^. Gender differences in health-reporting behaviour^21^ may play a role. However, the influence of sex on immunogenicity has been increasingly recognised for many vaccines^22,23^, including BCG^24,25^. Therefore, there may be a plausible biological sex difference in the occurrence of lymphadenopathy post vaccination.

Age-related differences in BCG lymphadenitis frequency amongst children have been reported, with lymphadenitis occurring more commonly in infants less than 6 months old, compared with older children and adults^1,8^. In our study, the lower risk of lymphadenopathy in older participants may be related to immunosenescence^26^. With increasing age, alterations in lymph node architecture (such as lymph node fibrosis) and declining node size occurs^27^. Consistent with this paradigm, in a recent COVID-19 vaccine trial, axillary swelling (suggesting lymphadenopathy) or tenderness was reported less frequently in adults older than 65 years old, compared with younger adults^28,29^.

There are some limitations to this study. First, our study was confined to adults; the clinical course of BCG abscess and lymphadenitis may differ in infants as the immune response varies with age^30,31^. Second, lymphadenopathy relied on self-palpation. However, those reporting nodes enlarged more than 1.5 cm in diameter and those with lymphadenopathy persisting more than two weeks had medical assessments arranged. Also, most participants were clinicians and all those reporting possible lymphadenopathy were contacted by an SMD. Third, data on the accuracy of intradermal vaccination techniques were not available for all participants. BCG strain and vaccination technique have been previously reported to influence the frequency of AEFI^1,4,6^.

Strengths include the use of active safety surveillance and the availability of safety data for 98% of participants, which allowed detailed evaluation of potential risk factors (both host- and vaccination-related) for AEFI. Review of clinical photographs helped the SMD assessment and follow-up.

In conclusion, we found that revaccination was associated with a higher risk of developing a BCG local adverse reaction than initial BCG vaccination. In those who developed an injection site abscess, this occurred earlier in revaccinated participants. Most local reactions self-resolved within 1 month. BCG-associated regional lymphadenopathy, but not injection site abscess, was influenced by age at vaccination and sex. In the low TB endemic setting of this study, a local adverse reaction was not attributable to LTBI. Our study has important implications for BCG vaccination policy in an era when BCG vaccination and revaccination is increasingly being considered for novel applications, including to reduce the impact of COVID-19^32^.

## Methods

### Setting and participants

Healthcare workers (HCW) were recruited in Stage 1 of the BRACE trial in six hospitals in Australia from March to May 2020, and randomised in a 1:1 ratio and open-label design to receive BCG vaccine or no BCG^32^. All participants received influenza vaccine to the contralateral arm within three days. Exclusion criteria comprised any contra-indication to BCG, including previous significant local BCG adverse reaction.

### Intervention

Participants randomised to BCG received a single dose of BCG-Denmark (AJ Vaccines, Copenhagen), 0.1 ml (corresponding to 2–8 × 10^5^ colony-forming units of *Mycobacterium bovis*, Danish strain 1331) intradermally in the left upper arm, using a short (10 mm) bevel needle (25 G to 30 G).

Three different BCG vaccine batches were used. BCG study vaccinators were classified as ‘experienced’ (previous experience in BCG clinics), ‘trained’ (trained in advance for the trial) or ‘learners’ (supervised training during the trial).

The administration was defined as ‘satisfactory’ when an intradermal bleb of 7 mm minimum diameter was documented after vaccination. Participants were informed about the normal expected local reaction to BCG vaccination and were instructed to contact study staff if they had any concerns.

### Data collection

Data were collected using REDCap^33^, including details on vaccine administration, and previous tuberculin skin tests (TST) and BCG vaccinations.

Information on vaccine site evolution and any subsequent lymphadenopathy were collected through participant-completed web-based daily questionnaires for 2 weeks post vaccination (vaccine diary) and at 3 months post vaccination (questionnaire). Serial vaccine site photographs were also collected. Additionally, participants could contact the investigators at any time after vaccination (via e-mail or telephone) if they had any concerns about their injection site.

### Active safety surveillance

Safety medical doctors (SMD) were trained for the BRACE trial to actively follow up any participant who reported a potential AEFI. Adverse events were recorded on standard forms. Photographs of potential injection site abscesses were reviewed by SMDs at regular quality and safety team meetings for consensus decision on classification. An interferon-gamma release assay (IGRA) test for latent tuberculosis infection (LTBI) was arranged 3 months after vaccination for participants with an injection site abscess.

### Case definitions

BCG injection site abscess was defined as a localised collection of pus, ≥1.5 cm in diameter at the injection site. BCG-associated lymphadenopathy was defined as palpable regional (axilla or neck) lymph node enlargement. Participants reporting lymphadenopathy ≥1.5 cm in diameter or persistent (>2 weeks duration) were recommended to seek medical assessment.

### Statistical analysis

Statistical analysis was done using StataIC 14.0 (Statacorp LP, College Station, TX, USA). The cumulative incidence of AEFI in the 3 months post-BCG vaccination was calculated among participants who provided vaccine safety data. Adverse events were compared between participants whose BCG vaccine was their first and those who had previously received BCG (‘revaccinated’) using Mann–Whitney and Chi-square test. Odds ratio (OR) and 95% confidence intervals (CI) were determined using univariate logistic regression. Significant factors (*p* value < 0.2) were included as possible covariates in a multivariate model. Backward stepwise exclusion of factors with *p* value > 0.05 was done to create the model.

Ethical approval was obtained from The Royal Children’s Hospital Human Research Ethics Committee (HREC 62586), with reciprocal ethics and governance approvals at each participating site. All participants provided signed informed consent prior to enrolment.

### Reporting Summary

Further information on research design is available in the Nature Research Reporting Summary linked to this article.

## Supplementary information

Supplementary Information

Reporting Summary

## Data Availability

The authors declare that the data supporting the findings of this study are available within the main tables and figures. All data are available from the corresponding author upon reasonable request.
