# Supplementary material for: Revaccination with Bacille Calmette-Guérin (BCG) is associated with an increased risk of abscess and lymphadenopathy
Source: NPJ Vaccines. 2022 Jan 14;7:6. doi: 10.1038/s41541-021-00421-5 (PMC8760267; doi:10.1038/s41541-021-00421-5)
Supplement: Supplementary file 1 — Supplementary Information [file 41541_2021_421_MOESM1_ESM.pdf]

VIC – MCRI, RCH

Name	Email	Role
**MCRI Central Team**		
**Nigel Curtis**	nigel.curtis@rch.org.au	BRACE Chief Principal Investigator
**Andrew Davidson**	Andrew.Davidson@rch.org.au	Investigator; Medical Director, MCTC
**Laure Pittet**	laure.pittet@mcri.edu.au	Investigator; BRACE Clinical Data Lead and Safety Lead
**Nicole Messina**	nicole.messina@mcri.edu.au	Investigator; BRACE Laboratory Lead
**Kirsten Perrett**	Kirsten.Perrett@rch.org.au	Investigator; Co-group Leader, Population Allergy; Clinician-Scientist Fellow, MCTC
**Amanda Gwee**	Amanda.Gwee@rch.org.au	Investigator; Team Leader, Infectious Group, MCRI
**Kaya Gardiner**	kaya.gardiner@mcri.edu.au	Investigator; former Program Manager
**Susan Perlen**	susan.perlen@mcri.edu.au	Current Program Manager
**Amber Sastry**	amber.sastry@mcri.edu.au	Former Program Manager
**Tenaya Jamieson**	tenaya.jamieson@mcri.edu.au	Senior Trial Coordinator
**Jia Wei Teo**	jiawei.teo@mcri.edu.au	Senior Trial Coordinator
**Thilanka Morawakage**	thilanka.morawakage@mcri.edu.au	Project Assistant
**Harriet Edmund**	harriet.edmund@mcri.edu.au	BRACE Communications Officer
**Biostatisticians**		
**Katherine Lee**	katherine.lee@mcri.edu.au	Senior Biostatistician
**Francesca Orsini**	francesca.orsini@mcri.edu.au	Biostatistician
**Cecilia Moore**	cecilia.moore@mcri.edu.au	Biostatistician
**Data Team**		
**Laure Pittet**	laure.pittet@mcri.edu.au	Investigator; Clinical Data Lead
**Ellie McDonald**	ellie.mcdonald@mcri.edu.au	Data Quality Senior Research Officer
**Richard Hall**	richard.hall@mcri.edu.au	Data Manager

**Casey Goodall**	casey.goodall@mcri.edu.au	Senior Data Officer
**Grace Gell**	grace.gell@mcri.edu.au	Data Follow-up Coordinator
**Nathan La**	nathan.la@mcri.edu.au	Data Quality RA
**Ann Krastev**	ann.krastev@mcri.edu.au	Data Quality RA
**Ross Dunn**	ross.dunn@mcri.edu.au	REDCap RA
**Luke Stevens**	luke.stevens@mcri.edu.au	Data Management Coordinator
**Nick McPhate**	nick.mcphate@mcri.edu.au	Original/Current BRACE Team
**Jack Ren**	jack.ren@mcri.edu.au	Start-up Data, Data Engineer, Gen V, MCRI
**Laboratory Team**		
**Nicole Messina**	nicole.messina@mcri.edu.au	Investigator; BRACE Laboratory Lead
**Rhian Bonnici**	rhian.bonnici@mcri.edu.au	Site Laboratory Coordinator
**Susie Germano**	susie.germaNo@mcri.edu.au	Laboratory RA
**Rebecca McElroy**	rebecca.mcelroy@mcri.edu.au	Laboratory RA
**Laboratory Scientists**		
**Ashleigh Wee-Hee**	ashleigh.weehee@mcri.edu.au	Regular Lab
**Ahmed Alamrousi**	ahmed.alamrousi@mcri.edu.au	Regular Lab
**Belinda Ortika**	belinda.ortika@mcri.edu.au	Regular Lab
**Casey Pell**	casey.pell@mcri.edu.au	Regular Lab
**Leena Spry**	leena.spry@mcri.edu.au	Regular Lab
**Kristy Azzopardi**	kristy.azzopardi@mcri.edu.au	Start-up Lab
**Nadia Mazarakis**	nadia.mazarakis@mcri.edu.au	Start-up Lab
**Ryan Toh**	zheng.quantoh@mcri.edu.au	Start-up Lab
**Vicki Bennett-Wood**	vicki.bennettwood@mcri.edu.au	Start-up Lab
**Sunitha Velagapudi**	sunitha.velagapudi@mcri.edu.au	Start-up Lab
**Jeremy Anderson**	jeremy.anderson@mcri.edu.au	Start-up Lab
**Amanda Vlahos**	amanda.vlahos@mcri.edu.au	Start-up Lab
**Anna Czajko**	anna.czajko@mcri.edu.au	Start-up Lab

**Biobanking**		
**Benjamin Ong**	benjamin.ong@mcri.edu.au	Lab Support; Head of Scientific Services
**Pedro Ramos**	pedro.ramos@mcri.edu.au	Biobanking Coordinator
**Anushka Karunanayake**	anushka.karunanayake@mcri.edu.au	Biobanking Staff
**Dinusha Gamage**	dinusha.gamage@mcri.edu.au	Biobanking Staff
**Enoshini Sooriyarachchi (Ushani)**	enoshini.sooriyarach@mcri.edu.au	Biobanking Staff
**Isabella Mezzetti**	isabella.mezzetti@mcri.edu.au	Biobanking Staff
**Karina De La Cruz**	karina.delacruz@mcri.edu.au	Biobanking Staff
**Ronita Singh**	ronita.singh@mcri.edu.au	Biobanking Staff
**Frances Oppedisano**	frances.oppedisano@mcri.edu.au	Laboratory Manager
**Study Visit and Phone call Staff**		
**Veronica Abruzzo**	veronica.abruzzo@mcri.edu.au	Visit coordinator; Senior Research Nurse Coordinator
**Joyce Chan**	joyce.chan@mcri.edu.au	Project Assistant
**Jac Cushnahan**	jac.cushnahan@mcri.edu.au	Clinic Staff
**Emma Burrell**	emma.burrell@mcri.edu.au	Clinic Staff
**Jamie Wedderburn**	jamie.wedderburn@mcri.edu.au	Clinic Staff
**Sarah Fowler**	sarah.fowler@mcri.edu.au	Clinic Staff
**Patricia Bimboese**	patricia.bimboese@rch.org.au	Clinic Staff; Start-up Support; Paediatric Trainee, RCH
**Hannah Elborough**	hannah.elborough@mcri.edu.au	Clinic Staff
**Jill Nguyen**	jill.nguyen@mcri.edu.au	Clinic Staff
**Stephanie Reynolds**	steph.reynolds@mcri.edu.au	Clinic Staff
**Liz O'Donnell**	liz.odonnell@mcri.edu.au	Clinic Staff
**Kirsty Bowes**	kirsty.bowes@mcri.edu.au	Clinic Staff
**Olivia Elkington**	oelkington@student.unimelb.edu.au	Clinic Staff
**Sam Macalister**	smacalister@student.unimelb.edu.au	Clinic Staff
**Catherine Flynn**	catherineflynn@hotmail.com.au	Clinic Staff
**Norine Ma**	norinem@student.unimelb.edu.au	Clinic Staff

**Bojana Gladanac**	bgladanac@student.unimelb.edu.au	Clinic Staff
**Morgan Bealing**	mbealing@student.unimelb.edu.au	Clinic Staff
**Isabelle Ooi**	wooi@student.unimelb.edu.au	Clinic Staff
**Nadia Olivier**	nadiaolivier94@gmail.com	Clinic Staff
**Monique Fernandez**	mfernandez1@student.unimelb.edu.au	Clinic Staff
**Angela Younes**	ayounes@student .unimelb.edu.au	Clinic Staff
**Kieran Fahey**	kfahey@student.unimelb.edu.au	Clinic Staff
**Lisa Shen**	lshen1@student.unimelb.edu.au	Clinic Staff
**Jesutofunmi Mojeed**	jmojeed@student.unimelb.edu.au	Clinic Staff
**Orygen Group**		Volunteers; phone calls to participants
**Pharmacy**		
**Donna Legge**	Donna.Legge@rch.org.au	Clinical Trial Pharmacist
**Annie Cobbledick**	Annie.cobbledick@rch.org.au	Pharmacist
**Kee Lim**	KeeLin.Lim@rch.org.au	Pharmacist
**Jo Cheah**	jo.cheah@rch.org.au	Pharmacist
**Jason Bell**	jason.bell@rch.org.au	Pharmacist
**Nurse Immuniser**		
**Sonja Elia**	sonja.elia@rch.org.au	Head of Immunisation Service
**Skye Miller**	Skye.Miller@rch.org.au	Vaccinator
**Lynne Addlem**	lynne.addlem@rch.org.au	Vaccinator
**Narelle Jenkins**	Narelle.Jenkins@rch.org.au	Vaccinator
**Nadine Henare**	Nadine.Henare@rch.org.au	Vaccinator
**Clare Brophy**	Clare.brophy@mcri.edu.au	Vaccinator
**Sigrid Pitkin**	sigrid.pitkin@mcri.edu.au	Vaccinator
**Anna Bourke**	anna.bourke@mcri.edu.au	Vaccinator
**Francesca Machingaifa**	Francesca.Machingaifa@mcri.edu.au	Vaccinator
**Kirsten Mitchell**	kirsten.mitchell@mcri.edu.au	Vaccinator

**Kate Wall**	Kate.wall@mcri.edu.au	Vaccinator
**Safety and Quality Monitoring**		
**Laure Pittet**	laure.pittet@mcri.edu.au	Investigator; Clinical Safety Lead
**Paola Villanueva**	Paola.Villanueva@rch.org.au	Safety Lead, PhD student
**Nigel Crawford**	nigel.crawford@mcri.edu.au	Director of SAEFVIC
**Wendy Norton**	Wendy.norton@mcri.edu.au	VIC Safety Representative
**Ushma Wadia**	ushma.Wadia@telethonkids.org.au	WA Safety Representative
**Alice Sawka**	alice.Sawka@sa.gov.au	SA Safety Representative
**Ketaki Sharma**	Ketaki.sharma@health.nsw.gov.au	NSW Safety Representative
**Darren Troeman**	D.P.R.Troeman@umcutrecht.nl	Former Netherland Safety Representative
**Cristina Prat Aymerich**	C.PratAymerich-2@umcutrecht.nl	Spain and Netherland Safety Representative
**Prof Adilia Warris**	A.Warris@exeter.ac.uk	UK Safety Representative
**Mariana Garcia Croda**	mgcroda@gmail.com	Brazil Safety Representative (Mato Grosso Do Sul)
**Jorge Rocha**	jorgeluiz.rocha@yahoo.com.br	Brazil Safety Representative (Rio de Janeiro)
**Bruno Jardim**	brunojardim89@hotmail.com	Brazil Safety Representative (Manaus)
**MCRI Start-up Team**		
**Carolyn Stewart**	carolyn.stewart@mcri.edu.au	Start-up Support; Business and Operations Manager, MCTC
**Katherine Lieschke**	katherine.lieschke@mcri.edu.au	Start-up Support; RCH Research Ethics and Governance, MCTC
**Jess Bucholc**	jess.bucholc@mcri.edu.au	Start-up Support Staff; coordination Monash
**Samantha Bannister**	Samantha.Bannister@rch.org.au	Start-up Support; PhD Student, Infectious Diseases Group, MCRI
**Eva Sudbury**	evasudbury@gmail.com	Start-up Support; PhD Student, Infectious Diseases Group, MCRI
**Emma Watts**	emma.watts@mcri.edu.au	Start-up Support; Regulatory Support
**Angela Young**	angela.young@mcri.edu.au	Start-up Support
**Chris Richards**	chris.richards@mcri.edu.au	Start-up Support Grants
**Christina Guo**	christina.guo@mcri.edu.au	Start-up Support; Paediatric Trainee, RCH
**Helen Thomson**	helen.thomson@mcri.edu.au	Support Grants; Research Manager, Asia-Pacific Health, MCRI
**Stephanie Firth**	stephanie.firth@mcri.edu.au	Start-up Support

**Marianna Ciaverella**	marianne.ciavarella@mcri.edu.au	Support Grants; Grants Manager, Grants Office, MCRI
**Matthew Hannan**	matthew.hannan@mcri.edu.au	Support Grants; Head of Engagement & Philanthropy, MCRI
**Erin Hill**	erin.hill@mcri.edu.au	Start-up Support; Population Allergy, MCRI
**Beatriz Camesella**	beatriz.camesellaper@mcri.edu.au	Start-up Support; Population Allergy, MCRI
**Ashleigh Rak**	ashleigh.rak@mcri.edu.au	Start-up Support Staff; coordination
**Sasha Odoi**	sasha.odoi@mcri.edu.au	Start-up Support Staff; coordination
**Megan Mathers**	megan.mathers@mcri.edu.au	Start-up Support Staff; coordination
**Sri Joshi**	sri.joshi@mcri.edu.au	Start-up support; Database Manager, MCTC
**Sophie Agius**	sophie.agius@mcri.edu.au	Start-up Support; logistics and consumables
**Alison Burns**	alison.burns@mcri.edu.au	Start-up Support; logistics and consumables
**Leah Steve**	leah.steve@mcri.edu.au	Start-up Support; logistics and consumables
**John Carlin**	john.carlin@mcri.edu.au	Director, Clinical Epidemiology & Biostatistics, MCRI

Name	Email	Role
**Steering Committee**		
**Andrew Steer**	andrew.steer@rch.org.au	Theme Director, Infection & Immunity, MCRI
**Ann Ginsberg**	ann.ginsberg@gatesfoundation.org	Deputy Director of TB Vaccines, Bill and Melinda Gates Foundation
**David Paterson**	d.paterson1@uq.edu.au	BRACE Steering Committee Chair
**Kanta Subbarao**	kanta.subbarao@influenzacentre.org	Director, WHO Collaborating Centre; Peter Doherty Institute
**Kim Mulholland**	kim.mulholland@mcri.edu.au	New Vaccines Group, MCRI
**Peter Richmond**	peter.richmond@uwa.edu.au	Principal Investigator WA, TKI
**Nigel Curtis**	nigel.curtis@rch.org.au	BRACE Chief Principal Investigator
**External Advisors**		
**Mihai Netea**	mihai.netea@radboudumc.nl	Head of Experimental Medicine, The Netherlands
**Richard Malley**	richard.malley@childrens.harvard.edu	Boston Children's Hospital; Harvard Medical School
**Adam Finn**	adam.finn@bristol.ac.uk	University of Bristol; NIHR Clinical Research Network; WHO
**Denise Faustman**	dfaustman@mgh.harvard.edu	Harvard University; Massachusetts General Hospital
**Frank Shann**	shannf@netspace.net.au	Start-up Support; Department of Paediatrics, University of Melbourne

**Kim Mulholland**	kim.mulholland@mcri.edu.au	New Vaccines Group, MCRI
**Regulatory, Legal and Finances**		
**Penny Glenn**	penny.glenn@mcri.edu.au	Senior Legal Counsel
**Laura Galletta**	laura.galletta@mcri.edu.au	Senior Study Coordinator, MCTC
**Amandine Philippart De Floy**	amandine.philippart@mcri.edu.au	Senior Contract Manager & Privacy Officer
**Neil Harker**	neil.harker@mcri.edu.au	Financial Consultant (Insurance & Risk)
**Karen Dalton**	karen.dalton@mcri.edu.au	Finance
**Galina Fidler**	galina.fidler@mcri.edu.au	Finance
**App Development**		
**Ivy Xie**	ivy@curvetomorrow.com.au	Product Manager, Curve Tomorrow
**Sandy Buchanan**	sandy@weguide.com.au	COO & Head of Partnerships at WeGuide
**Thijs Sondag**	thijs.sondag@mcri.edu.au	Innovation Consultant, Curve Tomorrow
**Media and Communications**		
**Harriet Edmund**	harriet.edmund@mcri.edu.au	BRACE Communications Officer
**Michelle Wearing-Smith**	michelle.wearingsmith@mcri.edu.au	Head of Communications & Marketing
**Tom Keeble**	tom.keeble@mcri.edu.au	Communications Manager
**Bridie Byrne**	bridie.byrne@mcri.edu.au	Communications Specialist
**Belle Ngien**	belle.ngien@mcri.edu.au	Marketing Projects Officer
**Fran Noonan**	fran.noonan@mcri.edu.au	Content Manager
**Logistics - Consumables**		
**Sophie Agius**	sophie.agius@mcri.edu.au	Start-up Support
**Alison Burns**	alison.burns@mcri.edu.au	Regular Stocks and Shipping
**Leah Steve**	leah.steve@mcri.edu.au	Regular Stocks and Shipping
**IT Support**		
**Wayne Mather**	wayne.mather@mcri.edu.au	CIO, MCRI
**Nick Evans**	nick.evans@mcri.edu.au	IT Infrastructure Manager, MCRI
**Luke Stevens**	luke.stevens@mcri.edu.au	REDCap expert

VIC – MONASH, EPWORTH

Name	Email	Role
**VIC Sites**		
**Niki Tan**	niki.tan@anaestheticservices.com.au	Epworth Healthcare PI
**Diane Dawson**	Di.Dawson@epworth.org.au	Site Coordinator
**Victoria Gordon**	victoria.gordon@epworth.org.au	Research Nurse
**Thilakavathi Chengodu**	thili.chengodu@epworth.org.au	Research Nurse
**Tony Korman**	tony.korman@monash.edu	Monash PI
**Jess O'Bryan**	Jessica.O'bryan@monashhealth.org	Site Coordinator

WA – PCH, FSH, SCGH

Name	Email	Role
**WA Sites**		
**Peter Richmond**	peter.richmond@uwa.edu.au	Principal Investigator WA, TKI, PCH
**Tobias Kollmann**	Tobias.Kollmann@telethonkids.org.au	Biosample and Data Use Committee WA Representative
**Ushma Wadia**	ushma.Wadia@telethonkids.org.au	WA Safety Representative
**Krist Ewe**	Yean.ewe@health.wa.gov.au	Sub-investigator
**Jaslyn Ong**	jaslyn.ong@health.wa.gov.au	Sub-investigator
**Joanne Ong**	Joanne.ong@telethonkids.org.au	Sub-investigator
**Andrea Meehan**	Andrea.meehan@health.wa.gov.au	Sub-investigator
**Carolyn Finucane**	Carolyn.finucane@telethonkids.org.au	Research Nurse

**Rachael Wallace**	Rachael.wallace@health.wa.gov.au	Research Nurse
**Annabelle Arnold**	Annabelle.arnold@health.wa.gov.au	Research Nurse (also helped at SCGH)
**Jemma Dunnill**	Jemma.dunnill@telethonkids.org.au	Research Nurse/follow up
**Catherine Power**	Catherine.power@health.wa.gov.au	Vaccinator
**Glady Perez**	Gladymar.perez@telethonkids.org.au	Vaccinator
**Jane Jones**	Jane.jones@telethonkids.org.au	Vaccinator/phlebotomist
**Camille Gibson**	Camille.gibson@telethonkids.org.au	Vaccinator/phlebotomist
**Fiona McDonald**	Fiona.mcdonald@telethonkids.org.au	Vaccinator/phlebotomist/follow up
**Lisa Stiglmayer**	Lisa.stiglmayer@health.wa.gov.au	Vaccinator
**Sally Rogers**	Sally.rogers@health.wa.gov	Vaccinator
**Lance Jarvis**	Lance.jarvis@health.wa.gov.au	Vaccinator
**Alexandra Truelove**	Alexandra.trulove@health.wa.gov.au	Vaccinator
**Jennifer Kent**	Jennifer.kent@telethonkids.org.au	Vaccinator
**Christina Anthony**	Christina.anthony@telethonkids.org.au	Phlebotomist
**Beth Arrowsmith**	Beth.arrowsmith@telethonkids.org.au	Phlebotomist
**Heidi Hutton**	Heidi.hutton@telethonkids.org.au	Phlebotomist
**Lorraine Flynn**	Lorraine.flynn@health.wa.gov.au	Phlebotomist
**Lisa Montgomery**	Lisa.montgomery@telethonkids.org.au	Phlebotomist/lab
**Jan Jones**	Jan.jones@telethonkids.org.au	Lab
**Nikki Schultz**	Nikki.Schultz@telethonkids.org.au	Lab
**Sonia McAlister**	Sonia.mcalister@telethonkids.org.au	Lab
**Sharon Clark**	Sharon.clark@telethonkids.org.au	Lab
**Kimberley Parkin**	Kimberley.parkin@telethonkids.org.au	Lab
**Holly Richmond**	holly.richmond@telethonkids.org.au	Follow up staff (part-time)/Site Coordinator contact
**Karen Jones**	Karen.a.jones@uwa.edu.au	Follow up staff
**Margaret Shave**	Margaret.shave@health.wa.gov.au	Pharmacist
**Melissa O’Donnell**	Melissa.o’donnell@health.wa.gov.au	Pharmacist

**Debbie Lalich**	Debbie.lalich@health.wa.gov.au	Pharmacist
**Justin Waring**	Justin.waring@health.wa.gov.au	Medical Director of the Anita Clayton Service- Follow up of the BCG vaccination adverse events of concern and also provision of nurses to train nurses regarding intradermal vaccination
**Laurens Manning**	laurens.manning@uwa.edu.au	Fiona Stanley Hospital PI
**Erin Latkovic**	erin.latkovic@health.wa.gov.au	Site Coordinator
**Michelle England**	michelle.england@health.wa.gov.au	FSH
**Michaela Lucas**	michaela.lucas@uwa.edu.au	Sir Charles Gairdner Hospital PI
**Susan Herrmann**	susan.herrmann@uwa.edu.au	Site Coordinator
**Hana Karuppasamy**	Hana.karuppasamy@health.wa.gov.au	Research Nurse
**Annabelle Arnold**	Annabelle.Arnold@health.wa.gov.au	Research Nurse
**Zaheerah Haywood**	zaheerah.haywood@health.wa.gov.au	Follow-up Staff
**Ruth Warren**	ruth.warren@uwa.edu.au	Follow-up Staff
**Nat Eiffler**	Nat.Eiffler@telethonkids.org.au	WA Participant Communication Support

SA – RAH, WCH

Name	Email	Role
**SA Sites**		
**Prof David Lynn**	David.lynn@sahmri.com	Principal Investigator SA, SAHMRI
**Prof Steve Wesselingh**	Steve.Wesselingh@sahmri.com	Sub Investigator
**Liddy Griffith**	liddy.griffith@sahmri.com	Senior Study Coordinator
**Domenic Sacca**	domenic.sacca@sahmri.com	Study Coordinator
**Angela Markow**	angela.markow@sahmri.com	Assistant Study Coordinator
**Rochelle Botten**	rochelle.botten@sahmri.com	Study Coordinator
**Miriam Lynn**	Miriam.Lynn@sahmri.com	Lab Researcher
**Stephen Blake**	stephen.blake@sahmri.com	Lab Researcher
**Natalie Stevens**	natalie.stevens@sahmri.com	Lab Researcher

**Georgina Eden**	Georgina.eden@sahmri.com	Lab Researcher
**Saoirse Benson**	Saoirse.benson@sahmri.com	Lab Researcher
**Tee Yee Chern**	Tee.Yee@sahmri.com	Lab Researcher
**Jane James**	jane.james@sahmri.com	Lab Researcher
**Dr Simone Barry**	Simone.barry@sa.gov.au	Royal Adelaide Hospital PI
**Catriona Doran**	Catriona.Doran@sa.gov.au	Site Coordinator
**Alice Sawka**	alice.Sawka@sa.gov.au	SA Safety Representative
**Prof Helen Marshall**	helen.marshall@adelaide.edu.au	Women's and Children's Hospital PI
**Christine Heath**	christine.heath@adelaide.edu.au	Site Coordinator
**Mark McMillan**	mark.mcmillan@adelaide.edu.au	Study Coordinator
**Meredith Krieg**	meredith.krieg@adelaide.edu.au	Research Nurse
**Mary Walker**	mary.walker@adelaide.edu.au	Research Nurse
**Louise Goodchild**	louise.goodchild@adelaide.edu.au	Research Nurse
**Dr Sue Evans**	sue.evans@adelaide.edu.au	Research Doctor

NSW – WC, WH, POWH, SCH, ST V'S

Name	Email	Role
**NSW Sites**		
**A/Prof Nick Wood**	nicholas.wood@health.nsw.gov.au	Principal Investigator NSW, WC
**Prof Craig Munns**	craig.munns@health.nsw.gov.au	Principal Investigator NSW, WC
**Aiken Dao**	aiken.dao@sydney.edu.au	Site Coordinator
**Katrina Sterling**	katrina.sterling@health.nsw.gov.au	Site Coordinator (WC, POWH, SCH)
**Lisa Pelayo**	lisa.pelayo@health.nsw.gov.au	NSW State Coordinator
**Andrew Dunn**	Andrew.Dunn@health.nsw.gov.au	Clinic Staff
**Therese Baulman**	therese.baulman@health.nsw.gov.au	Vaccinator
**Mary Corbett**	mary.corbett@health.nsw.gov.au	Vaccinator

**Maria Desylva**	maree.desylva@health.nsw.gov.au	Vaccinator
**Rosemary Joyce**	rosemary.joyce@health.nsw.gov.au	Clinical Nurse Consultant
**Evangeline Gardiner**	Evangeline.Gardiner@health.nsw.gov.au	Research Assistant
**A/Prof Mark Douglas**	mark.douglas@sydney.edu.au	Westmead Hospital PI
**Clinton Colaco**	Clinton.colaco@health.nsw.gov.au	Sub-investigator
**Kate Hamilton**	Kate.Hamilton@health.nsw.gov.au	Site Coordinator
**Dr Brendan McMullan**	brendan.mcmullan@health.nsw.gov.au	Sydney Children's Hospital, Randwick PI
**Pamela Palasanthiran**	pamela.palasanthiran@health.nsw.gov.au	Clinic Staff
**Adam Bartlett**	adam.bartlett@health.nsw.gov.au	Clinic Staff
**Phoebe Williams**	phoebe.williams1@health.nsw.gov.au	Clinic Staff
**A/Prof Jeffrey Post**	jeffrey.post@health.nsw.gov.au	Prince of Wales Hospital PI
**Renier Lagunday**	Renier.Lagunday@health.nsw.gov.au	Clinic Staff
**Justin Beardsley**	Justin.beardsley@health.nsw.gov.au	Clinic Staff
**Kristen Overton**	Kristen.overton@health.nsw.gov.au	Clinic Staff
**Nikki Bergant**	nikki.bergant6@gmail.com	Vaccinator
**Dr Anthony Byrne**	anthony.byrne@svha.org.au	St Vincent's Hospital Sydney PI
**Lee Mead**	Lee.Mead@svha.org.au	Site Coordinator
**Yasmeen Al-Hindawi**	Yasmeen.Al-hindawi@svha.org.au	Site Investigator SVHS
**Sarah Barney**	Sarah.Barney@svha.org.au	Clinical Trial Assistant
**Nikki Bergant**	nikki.bergant6@gmail.com	Vaccinator
**Dr Rama Kandasamy**	Rama.Kandasamy@health.nsw.gov.au	Key Contact
**Twinkle Bahaduri**	twinkle.bahaduri@health.nsw.gov.au	NSW Ethics/Governance Submission Coordinator
**Ketaki Sharma**	Ketaki.sharma@health.nsw.gov.au	NSW Safety Representative

THE NETHERLANDS

Name	Email	Role
**UMC Utrecht**		
**Prof Marc Bonten**	m.j.m.bonten@umcutrecht.nl	Sponsor Investigator - Europe PI
**Leo Van Den Heuvel**	L.M.C.v.denHeuvel-2@umcutrecht.nl	
**Cristina Prat Aymerich**	C.PratAymerich-2@umcutrecht.nl	Coordinating Investigator - Europe
**Nicolette van Sluis**	N.L.J.vanSluis@umcutrecht.nl	Project Manager European Projects
**Astrid Suiker**	astrid.suiker@juliusclinical.com	Clinical Research Associate
**Radhika Ganpat**	j.r.ganpat@umcutrecht.nl	Clinical Trial Assistant
**Marije van der Waal**	M.vanderWaal-13@umcutrecht.nl	Junior Program Manager (Spain)
**Sigrid van der Veen**	s.vanderveen-11@umcutrecht.nl	Junior Program Manager (Dutch)
**Engelien Septer-Bijleveld**	engelien.septerbijleveld@juliusclinical.com	Sr. Project Manager
**Titia Leurink**	titia.leurink@juliusclinical.com	Jr. Project Manager
**Chantal van de Ven**	chantal.vandeven@juliusclinical.com	Clinical Trial Assistant
**Axel Janssen**	a.b.janssen-9@umcutrecht.nl	Head of Central Laboratory
**Beatrijs Wolters**	b.e.wolters-2@umcutrecht.nl	
**Darren Troeman**	D.P.R.Troeman@umcutrecht.nl	Medical Monitor
**Toos Lemmers**	A.D.P.Lemmens-2@umcutrecht.nl	Study Coordinator
**Endriaen Prajitno**	E.Prajitno@umcutrecht.nl	Pharmacist
**Anne Boon**	A.Boon@umcutrecht.nl	Laboratory
**Marjoleine van Opdorp**	M.J.W.vanOpdorp@umcutrecht.nl	Research Nurse
**Amphia Hospital**		
**Prof Jan Kluytmans**	jankluytmans@gmail.com	Hospital PI
**Dr M.M.L (Miranda) van Rijen**	MVanRijen@amphia.nl	Sub Investigator
**Dr Wouter Bijllaardt**	WBijllaardt@amphia.nl	Sub Investigator
**Linda van Mook**	Lmlresearch@amphia.nl; Lvanmook@amphia.nl	Study Coordinator

**Jannie Romme**	Lmlresearch@amphia.nl; JRomme@amphia.nl	Study Coordinator
**H. van Onzenoort**	trialbureau@amphia.nl	Pharmacist
**Vivian Zwart**	vzwart@amphia.nl	Vaccinator
**Rijnstate Hospital**		
**Jet Gisolf**	JGisolf@rijnstate.nl	Hospital PI
**Dr Robert Jan Hassing**	RHassing@rijnstate.nl	Sub Investigator
**Harald Verheij**	HVerheij@rijnstate.nl	Study Coordinator
**P.M.G. Filius**	trialapotheek@rijnstate.nl	Pharmacist
**Frances Greven**	Fgreven@rijnstate.nl	Research Nurse
**Lieke Preijers**	LPreijers@rijnstate.nl	Research Nurse
**P.M.Verhoeven**	pverhoeven@rijnstate.nl	Research Nurse
**J. H. van Leusen**	jvanleusen@rijnstate.nl	Vaccinator
**R. C. Pon**	rpon@rijnstate.nl	Vaccinator
**Sille Pelser**	spelser@rijnstate.nl	Research Nurse
**Marlot Uffing**	muffing@rijnstate.nl	Research Nurse
**Danique Huijbens**	Dhuijbens@rijnstate.nl	Research Nurse
**Noord West Ziekenhuis**		
**Dr Wim Boersma**	w.boersma@nwz.nl	Hospital PI
**Dr Nienke Paternotte**	n.paternotte@nwz.nl	Sub Investigator
**Lida Stooper**	researchlongziekten@nwz.nl	Study Coordinator
**Anke Rol**	researchlongziekten@nwz.nl	Study Coordinator
**Paul Kloeg**	p.kloeg@nwz.nl	Pharmacist
**Khanh Nguyen**	k.nguyen@nwz.nl	Pharmacy Assistant
**Kitty Molenaar**	c.a.m.molenaar-groot@nwz.nl	Pharmacy Coordinator
**Radboud UMC**		
**Dr Jaap ten Oever**	Jaap.tenOever@radboudumc.nl	Hospital PI
**Dr Simone Moorlag**	Simone.Moorlag@radboudumc.nl	Sub Investigator

**Dr Esther Taks**	Esther.Taks@radboudumc.nl	Sub Investigator
**Prof Mihai Netea**	Mihai.Netea@radboudumc.nl	Sub Investigator
**Rob ter Heine**	R.terHeine@radboudumc.nl	Pharmacist
**Helga Dijkstra**	Helga.Dijkstra@radboudumc.nl	Laboratory
**St Antonius Hospital**		
**Dr Bob Meek**	b.meek@antoniusziekenhuis.nl	Hospital PI
**Dr Nienke Roescher**	n.roescher@antoniusziekenhuis.nl	Sub Investigator
**Kitty Blauwendraat**	k.blauwendraat@antoniusziekenhuis.nl	Study Coordinator
**Carmen Zhou**	c.zhou@antoniusziekenhuis.nl	SC/Vaccinator
**Houda Harbech**	h.harbech@antoniusziekenhuis.nl	SC/Vaccinator
**Menno te Riele**	m.te.riele@antoniusziekenhuis.nl	SC/Vaccinator

SPAIN

Name	Email	Role
**Mutua Terrassa University Hospital**		
**Tomás Perez Porcuna**	tomasperez@mutuaterrassa.es	Hospital PI
**Dr Esther Calbo**	ecalbo@mutuaterrassa.es	Sub Investigator
**Cristina Badia Marti**	cbadia@mutuaterrassa.cat	Study Coordinator
**Susana Gonzalez Marcos**		Sub Investigator
**Sonia Sallent**	ssallent@mutuaterrassa.es	Study Nurse
**Maria Roser Font**	rfont@mutuaterrassa.es	Study Nurse
**University Hospital Germans Trias I Pujol**		
**Dr Antoni Rosell**	arosellg.germanstrias@gencat.cat	Hospital PI
**Adrian Siles Baena**	asiles@igtp.cat	Pharmacist
**Jose Dominguez**	jadomb69@gmail.com	Laboratory
**Ana Maria Barriocanal**	ambarriocanal@igtp.cat	Study Coordinator

**Alicia Lacoma**	alacoma@igtp.cat	Study Coordinator
**Maria Esteve**	mariaesteve.germanstrias@gencat.cat	Researcher
**Irma Casas**	icasas.germanstrias@gencat.cat	Researcher
**Guillermo Mena**	guillemena.germanstrias@gencat.cat	Researcher
**Anabel Barriocanal**	ambarriocanal@igtp.cat	Researcher
**Sandra Vidal**	svidal@igtp.cat	Research Nurse
**Gemma Molina**	gmolina@igtp.cat	Researcher
**Irene Latorre**	ilatorre@igtp.cat	Researcher
**Barbara Molina**	bmolina@igtp.cat	Researcher
**Raquel Villar**	rvillar@igtp.cat	Researcher
**Ester Valls**		Researcher
**Patricia Comella**		Researcher
**University Hospital Virgen Macarena**		
**Prof Jesús Rodríguez-Baño**	jesusrb@us.es	Hospital PI
**Lydia Barrera**	redancraid.hvm.sspa@juntadeandalucia.es	Study Coordinator
**Enriqueta Tristán**	redancraid.hvm.sspa@juntadeandalucia.es	Study Coordinator
**Jose Manuel Carrerero**	ensayosclinicosmacarena@yahoo.es	Pharmacist
**Carlos García**	administracion.eecc.hvm.sspa@juntadeandalucia.es	Clinical Trials Management
**Almudena de la Serna**	almudena.serna@juntadeandalucia.es	Clinical Trials Management
**Virginia Palomo Jiménez**	virgi2015@hotmail.com	Study Coordinator
**Angel Dominguez Castellano**	adomin60@gmail.com	Sub Investigator
**Maria Jose Rios Vilegas**	mjriosvillegas@gmail.com	Sub Investigator
**Reyes Lopez Marques**	mreyes.lopez.sspa@juntadeandalucia.es	Sub Investigator
**Nicolas Navarrette**	alambrada21@gmail.com	Sub Investigator
**Maria Dolores del Toro Lopez**	mdeltoro@us.es	Sub Investigator
**Rosario Vigo Ortega**	charovo2102@gmail.com	Research Nurse
**David Gutierrez Campos**	david.gutierrezcampos@hotmail.com	Research Nurse

**Ana Belen Martin Gutierrez**	anab.martin.gutierrez@outlook.com	Research Nurse
**Marie-Alix Clement Espindola**	mariealixclement@gmail.com	Research Nurse
**Encarnacion Ramirez de Arellano**	encarnacion.ramirezarellano.sspa@juntadeandalucia.es	Laboratory
**Alvaro Pascual Hernandez**		Laboratory
**Maria Carmen Roque**	mariac.roque@juntadeandalucia.es	Scientific Coordinator
**Teresa Rodrigues**	tereler@gmail.com	Laboratory
**Raquel Serrano**	ryrserrano@gmail.com	Laboratory
**Bouchra Daitiri**		Pharmacist
**Ines Portillo Calderón**	ines6290@gmail.com	Laboratory
**Natalia Bustos**	natalia.bustos@juntadeandalucia.es	Study Coordinator
**Rocio del Alba Rey Morillo**	rocio.rey@juntadeandalucia.es	Clinical Research Associate
**University Hospital Cruces**		
**Dr. Josune Goikoetxea**	ANEJOSUNE.GOIKOETXEAAGIRRE@osakidetza.eus	Hospital PI
**Atsegiñe Cangas**	ATSEGINE.CANGAGARCES@osakidetza.eus	Study Coordinator
**Cristina Perez**		Pharmacist
**Leticia Jorge**	ensayosclinicos.farmaciacruces@osakidetza.eus	Pharmacist
**Erika Castro**	ERIKA.CASTROAMO@osakidetza.eus	Laboratory
**Raquel Coya**	RAQUEL.COYAGUERRERO@osakidetza.eus	Laboratory
**Marqués de Valdecilla University Hospital**		
**Dr María Carmen Fariñ Álvarez**	mcarmen.farinas@scsalud.es	Hospital PI
**Dr Francisco Arnaiz de las Revillas Almajano**	francisco.arnaizlasrevillas@scsalud.es	Sub Investigator
**Claudia González Rico**	claudia.glez.rico@gmail.com; claudia.gonzalez@scsalud.es	Site Coordinator
**Dr Teresa Giménez Poderos**	farmacia_ensayos.humv@scsalud.es	Pharmacist
**Dr Jorge Calvo Montes**	jorge.calvo@scsalud.es	Laboratory
**Olga Valero**	olga.valero@scsalud.es	Study Nurse
**Noelia Vega**	noelia.vega@scsalud.es	Study Coordinator
**Nuria Sanchez**		Study Nurse

**Blanca Sanchez**		Study Coordinator
**Pilar Bohedo Garcia**	mpilar.bohedo@scsalud.es	Vaccinator
**Manuel Gutierrez Cuadra**	manuel.gutierrezc@scsalud.es	Sub Investigator

THE UK

Name	Email	Role
**University of Exeter/Exeter Clinical Trials Unit**		
**Prof John Campbell**	john.campbell@exeter.ac.uk	Principal Investigator (UK)
**Prof Adilia Warris**	A.Warris@exeter.ac.uk	Co-Principal Investigator (UK)
**Shelley Rhodes**	S.Rhodes@exeter.ac.uk	Senior Trial Manager
**Lynne Quinn**	L.Quinn@exeter.ac.uk	ExeCTU Director of Operations
**Abby O'Connell**	a.j.oconnell@exeter.ac.uk	Trial Manager
**Emily Fletcher**	E.Fletcher@exeter.ac.uk	Trial Manager
**Amy McAndrews**	A.McAndrew@exeter.ac.uk	Trial Manager
**Bethany Whale**	B.Whale@exeter.ac.uk	Trial Coordinator
**Harry Tripp**	H.E.Tripp@exeter.ac.uk	Data Manager
**Rosie Owens**	R.S.Owens@exeter.ac.uk	Data Manager
**Liam Fouracre**	L.Fouracre@exeter.ac.uk	Data Manager
**Phoebe Dawe**	P.Dawe@exeter.ac.uk	Data Manager
**Jakob Onysk**	jao208@exeter.ac.uk	Data Manager
**Helen Catterick**	helen.catterick@nhs.net	Safety Medical Doctor
**Lorrie Symons**	lorrie.symons@nhs.net	Safety Medical Doctor
**Georgina Newman**	gn272@exeter.ac.uk	Safety Medical Doctor
**Alison Gifford**	ag839@exeter.ac.uk	Safety Medical Doctor
**Clare Seamark**	clare.seamark@nhs.net	Safety Medical Doctor
**David Seamark**	david.seamark@nhs.net	Safety Medical Doctor

**Christopher Martin**	C.Martin4@exeter.ac.uk	Pharmacist
**Marcus Mitchell**	M.R.P.Mitchell@exeter.ac.uk	Laboratory Technician
**Louise Vennells**	L.Vennells@exeter.ac.uk	Senior Press and Media Manager
**St Leonard's Practice**		
**Dr Alex Harding**	a.m.harding@nhs.net	Practice PI
**Gemma Lockhart**	gemma.lockhart@nhs.net	Nurse
**Kate Sidaway-Lee**	k.sidaway-lee@nhs.net	Research Fellow
**Ide Lane Surgery**		
**Dr Daniel Webber-Rookes**	d.webber-rookers@nhs.net	Practice PI
**Sarah Manton**	sarahmanton@nhs.net	Nurse
**Dr Sam Hilton**	sam.hilton@nhs.net	Clinician
**Travel Clinic**		
**James Moore**	james@travelhealthconsultancy.co.uk	Clinic PI/Nurse
**Royal Devon and Exeter NHS Foundation Trust**		
**Dr Michael Gibbons**	michael.gibbons2@nhs.net	Hospital PI
**Josephine Studham**	j.m.studham@exeter.ac.uk	NIHR Exeter Clinical Research Facility Manager
**Bridget Knight**	B.A.Knight@exeter.ac.uk	Research Nurse
**Julie Moss**	Julie.moss10@nhs.net	Research Nurse
**Sarah Statton**	s.statton@nhs.net	Clinician
**Glendevon Medical Practice**		
**Tamsin Venton**	Tamsin.venton@nhs.net	Practice PI
**Will Moyle**	will.moyle@nhs.net	Practice Manager
**Robert Harrison**	robert.harrison2@nhs.net	GP
**Rachel Dixon**	rdixon3@nhs.net	GP
**Lydia Hall**	lydiahall@nhs.net	Research Practioner
**Jill Fairweather**	jill.fairweather@nhs.net	Practice Nurse

Name	Email	Role
**UEMS, Mato Grosso do Sul**		
**Julio Croda**	juliocroda@gmail.com	Principal Investigator (MS)
**Prof Roberto Oliveira**	roberto@uems.br; prof.roberto@me.com	Study Coordinator
**Patricia Vieira**	patriciavieira.s@hotmail.com	Study Coordinator
**Daniel Tsuha**	danlnca@gmail.com	Data Manager
**Marco Puga**	marco.m.puga@gmail.com	Laboratory Lead
**Bruna Tayara Leopoldina Meireles**	bruna.tayara@hotmail.com	Operator not blind
**Carolinne Abreu**	abreucarolinne5@gmail.com	Operator not blind
**Dyenyffer Stéffany Leopoldina dos Santos**	dyenyffer98@hotmail.com	Operator not blind
**Miriam de Jesus Costa**	myricosta29@gmail.com	Operator not blind
**Caroliny Veron Ramos**	carolinyvr19@gmail.com	Telephonist
**Claudinalva Ribeiro dos Santos**	claudinalva2009@gmail.com	Telephonist
**Guilherme Teodoro de Lima**	guilhermexaira@gmail.com	Telephonist
**Katya Martinez Almeida**	katyamartinez1982@gmail.com	Telephonist
**Matheus Machado Ramos**	mrmachadobiotec@gmail.com	Telephonist
**Wellyngthon Espindola Ayala**	wellyngtonespindola@gmail.com	Telephonist
**Bianca Maria Silva Menezes Arruda**	biancamenezes14@hotmail.com	Laboratory
**Camila Bitencourt de Andrade**	camilabitencourtdeandrade@gmail.com	Laboratory
**Débora dos Santos Silva**	d.rodruigues85@bol.com.br	Laboratory
**Joyce dos Santos Lencina**	joycedslencina@gmail.com	Laboratory
**Lais Alves da Cruz**	laismcr@outlook.com	Laboratory
**Mariana Mayumi Tadokoro**	mayumitadokoro@gmail.com	Laboratory
**Paulo Victor Rocha da Silva**	pvitor993@gmail.com	Laboratory
**Karla Regina Warszawski de Oliveira**	karlareginawo@gmail.com	Pharmaceutical Head

**Andrea Antonia Souza de Almeida dos Reis Pereira**	farmaceuticaandreareis@gmail.com	Pharmaceutical
**Iara Rodrigues Fernandes**	iara_rfernandes@yahoo.com.br	Pharmaceutical
**Roberta Carolina Pereira Diogo**	roberta_rcpd@hotmail.com	Pharmaceutical
**Rodrigo Cezar Dutra Escobar**	rodrigo.escoba@outlook.com	Pharmaceutical
**Adelita Agripina Refosco Barbosa**	adelita.barbosa@hotmail.com	Nurse
**Adriely de Oliveira**	adriely.o.b@gmail.com	Nurse
**Felipe Zampieri Vieira Batista**	lipe_zampieri@hotmail.com	Nurse
**Hugo Miguel Ramos Vieira**	hugobossram@hotmail.com	Nurse
**Jhenyfer Thalyta Campos Angelo**	jhenyfer_thalyta@hotmail.com	Nurse
**Karla Lopes dos Santos**	karla.lds@hotmail.com	Nurse
**Leticia Ramires Figueiredo**	le.enf.ramires@gmail.com	Nurse
**Lilian Batista Silva Muranaka**	liabatistamuranaka@gmail.com	Nurse
**Thaynara Haynara Souza da Rosa**	thayouza@gmail.com	Nurse
**Fábio Mauricio Nogueira Gomes**	fabinhomng@hotmail.com	Driver
**Leandro Galdino Cavalcanti Gonçalves**	leandrogcg@gmail.com	Driver
**Mariana Garcia Croda**	mgcroda@gmail.com	Safety Monitoring
**Matheus Vieira de Oliveira**	vieiramatheus79@gmail.com	Data Manager
**Mayara Góes dos Santos**	goes_may@hotmail.com	Coordinator Assistant
**Fabiani de Morais Batista**	fabianimb@hotmail.com	Coordinator Assistant

BRAZIL – RIO DE JANEIRO

Name	Email	Role
**Rio de Janeiro**		
**Margareth Dalcolmo**	margarethdalcolmo@gmail.com	Principal Investigator (Rio)
**Glauce Dos Santos**	glaucedossantos@gmail.com	Study Coordinator
**Ana Paula Conceição de Souza**	souzaannadi@hotmail.com	Assistant Coordinator

**Ivan Maia**	ivanramosnut@gmail.com	Data Manager
**Adriano Gomes**	adriano.gomes@ini.fiocruz.br	Laboratory Lead
**Alda Cruz**	alda@ioc.fiocruz.br	Laboratory Lead
**Samyra Almeida Da Silveira**	samybiologia@gmail.com	Laboratory RA
**Maria Luciana Silva De Freitas**	mluciana@id.uff.br	Laboratory RA
**Rosa Maria Plácido Pereira**	rosamplacido@gmail.com	Laboratory RA
**Gabriela Correa E Castro**	gabi.castro027@gmail.com	Laboratory RA
**Paulo Leandro Garcia Meireles Junior**	paulolgmj@gmail.com	Laboratory RA
**Erica Fernandes Silva**	ericafernandesfarma@gmail.com	Pharmacist
**Aline Gerhardt de Oliveira**	agerhardt@ensp.fiocruz.br	Pharmacist
**Cristiane Machado**	cmarcondes07@gmail.com	Pharmacist
**Jorge Rocha**	jorgeluiz.rocha@yahoo.com.br	Safety Lead
**Ligia Olivio**	ligiamon@gmail.com	Safety Medical Doctor
**Estela Carvalho**	estellacarvalho@gmail.com	Safety Medical Doctor
**Telma Goldenberg**	telmagold@gmail.com	Safety Medical Doctor
**Simone Collopy**	sicollopy@gmail.com	Safety Medical Doctor
**Girlene Pandine**	gspandine@gmail.com	Phlebotomist
**Rafaela Silva**	rafaelaenf@yahoo.com.br	Phlebotomist
**Daniella Mesquita**	danielle.mesquita@gmail.com	Phlebotomist
**Cíntia Maria Lopes Alves**	cintiamarialopes9@gmail.com	Phlebotomist
**Ana Rita Lopes Souza**	nanalopes_18@hotmail.com	Call Operator
**Marilena Oliveira**	marilena_pires@hotmail.com	Call Operator
**Cíntia Lopes Bogéa**	bogeacintia@gmail.com	Phlebotomist
**Marciléia Soares D.Allão Chaves**	marcileia.enf@gmail.com	Study Nurse
**Ayla Alcoforado da Silva dos Santos**	aylauanny@gmail.com	Study Nurse
**Renato da Costa Silva**	renatocostta1212@gmail.com	Study Nurse
**Marilda Siqueira**	mmsiq@ioc.fiocruz.br	Virology Laboratory Lead

Name	Email	Role
**Manaus**		
**Marcus Lacerda**	marcuslacerda.br@gmail.com	Principal Investigator (Manaus)
**Bruno Jardim**	brunojardim89@hotmail.com	Co-Principal Investigator
**Mariana Simão**	marianasimaoxavier@gmail.com	Study Coordinator
**Fernando Val**	ffaval@gmail.com	Study Coordinator
**Larissa Brasil**	larissa_brasil@hotmail.com	Laboratory Head
**Christiane Prado**	christianeprado95@gmail.com	Nurse Leader
**Kelry Mazurega**	kelryoliveira27@gmail.com	Pharmacist Leader
**Vanderson Sampaio**	vandersons@gmail.com	Data Manager
**Anna Gabriela Santos**	annagabrielarezende@gmail.com	Call Center Head
**Tyane Jardim**	tyane_almeida@hotmail.com	Safety Medical Doctor
**Bernardo Maia**	bernardo.mpesq88@gmail.com	Data Assistant
**Ariandra Sartim**	ariandrag@hotmail.com	Pharmacist
**Alexandre Trindade**	atrindade15@gmail.com	Pharmacist
**Rosangela Melo**	tanantasmelo@gmail.com	Pharmacist
**Arthur Otsuka**	arthurfarmaceutico@outlook.com	Pharmacist
**Dayanne Barros**	dayanne.barroslk@gmail.com	Pharmacist
**Ana Carolina Furtado**	acazevedofurtado@gmail.com	Call Operator
**Rayssa Paes**	Rayssapaesv2@gmail.com	Call Operator
**Ramon Castro**	rmnpeixoto@outlook.com	Call Operator
**Ana Greyce Capella**	Ana.g.capella@gmail.com	Call Operator
**Daniel Santos**	elguedes07@gmail.com	Call Operator
**Erlane Costa**	erlanesantiago72854@gmail.com	Call Operator
**Larissa Gama**	Larissamotag@gmail.com	Call Operator

**Maria Gabriela Oliveira**	Mgvdo.enf16@uea.edu.br	Call Operator
**Thamires Freitas**	mesquita.thamires24@gmail.com	Call Operator
**Antonny Sousa**	michaelantonny@gmail.com	Data quality control
**Thais Oliveira**	tgo.enf19@gmail.com	Nurse
**Juliana Silva**	julianansilva22@gmail.com	Nurse
**Adria Vasconcelos**	adria.lemos22@gmail.com	Nurse
**Joel Junior**	joeljuniorifpa@gmail.com	Nurse
**Elizandra Nascimento**	eliizandrafn@gmail.com	Nurse
**Tilza Santos**	thilzadepaula@gmail.com	Nurse
**Evelyn Queiroz**	Evelyn.queiroz24@hotmail.com	Nurse
**Handerson Pereira**	handersonsilva13@hotmail.com	Nurse
**Laleyska Rodrigues**	Laleyska@gmail.com	Nurse
**Fabiane Bianca Barbosa**	bianca.albar11@gmail.com	Laboratory
**Juliana Neves**	neves.juh.costa@gmail.com	Laboratory
**Emanuelle Silva**	emanuellelira96@gmail.com	Laboratory
**Adriana Marins**	dricka.ferreira16@hotmail.com	Laboratory
**Bebeto Rodrigues**	b.rodrigues1403@gmail.com	Laboratory
**Paulo Henrique Andrade**	andrade.biomedi@gmail.com	Laboratory
**Ingrid Oliveira**	ingrid.igor30@gmail.com	Laboratory
**Gabrielle Pereira**	gabriellepereira61@gmail.com	Laboratory
**Vanessa Godinho**	vanessa.vk.castro@gmail.com	Laboratory
