# Supplementary material for: Revaccination with Bacille Calmette-Guérin (BCG) is associated with an increased risk of abscess and lymphadenopathy
Source: NPJ Vaccines. 2022 Jan 14;7:6. doi: 10.1038/s41541-021-00421-5 (PMC8760267; doi:10.1038/s41541-021-00421-5)
Supplement: Supplementary file 2 — Reporting Summary [file 41541_2021_421_MOESM2_ESM.pdf]

nature portfolio

Corresponding author(s): DBPR NPJVACCINES-01602

Last updated by author(s): Sep 13, 2021

Reporting Summary

Nature Portfolio wishes to improve the reproducibility of the work that we publish. This form provides structure for consistency and transparency in reporting. For further information on Nature Portfolio policies, see our Editorial Policies and the Editorial Policy Checklist.

Statistics

For all statistical analyses, confirm that the following items are present in the figure legend, table legend, main text, or Methods section.

n/a	Confirmed	
☐	☒	The exact sample size (*n*) for each experimental group/condition, given as a discrete number and unit of measurement
☐	☒	A statement on whether measurements were taken from distinct samples or whether the same sample was measured repeatedly
☐	☒	The statistical test(s) used AND whether they are one- or two-sided<br>*Only common tests should be described solely by name; describe more complex techniques in the Methods section.*
☐	☒	A description of all covariates tested
☐	☒	A description of any assumptions or corrections, such as tests of normality and adjustment for multiple comparisons
☐	☒	A full description of the statistical parameters including central tendency (e.g. means) or other basic estimates (e.g. regression coefficient) AND variation (e.g. standard deviation) or associated estimates of uncertainty (e.g. confidence intervals)
☐	☒	For null hypothesis testing, the test statistic (e.g. *F*, *t*, *r*) with confidence intervals, effect sizes, degrees of freedom and *P* value noted<br>*Give P values as exact values whenever suitable.*
☒	☐	For Bayesian analysis, information on the choice of priors and Markov chain Monte Carlo settings
☒	☐	For hierarchical and complex designs, identification of the appropriate level for tests and full reporting of outcomes
☐	☒	Estimates of effect sizes (e.g. Cohen's *d*, Pearson's *r*), indicating how they were calculated

Our web collection on statistics for biologists contains articles on many of the points above.

Software and code

Policy information about availability of computer code

Data collection	Research electronic data capture (REDCap) is a secure web application for building and managing online surveys and databases.
Data analysis	Statistical analysis was done using StataIC 14.0 (Statacorp LP, College Station, TX, USA).

For manuscripts utilizing custom algorithms or software that are central to the research but not yet described in published literature, software must be made available to editors and reviewers. We strongly encourage code deposition in a community repository (e.g. GitHub). See the Nature Portfolio guidelines for submitting code & software for further information.

Data

Policy information about availability of data

All manuscripts must include a data availability statement. This statement should provide the following information, where applicable:

- Accession codes, unique identifiers, or web links for publicly available datasets
- A description of any restrictions on data availability
- For clinical datasets or third party data, please ensure that the statement adheres to our policy

The authors declare that the data supporting the findings of this study are available within the main tables and figures. All data are available from the corresponding author upon reasonable request.

Field-specific reporting

Please select the one below that is the best fit for your research. If you are not sure, read the appropriate sections before making your selection.

☒ Life sciences ☐ Behavioural & social sciences ☐ Ecological, evolutionary & environmental sciences

For a reference copy of the document with all sections, see nature.com/documents/nr-reporting-summary-flat.pdf

Life sciences study design

All studies must disclose on these points even when the disclosure is negative.

Sample size	Active surveillance data from a multicentre randomised controlled trial of BCG vaccination to reduce the impact of COVID-19 in healthcare workers (the BRACE trial) was used. Data was used from all participants who received the BCG vaccine in BRACE trial Stage 1, and who provided vaccine safety data. The sample size is sufficient for the aims of this study, when compared to previous studies that report incidence of BCG-related adverse reactions (Table 3 in manuscript).
Data exclusions	Of 1415 participants who received the BCG vaccine in BRACE trial Stage 1, 28 participants were excluded, as they did not provide vaccine safety data (did not complete vaccine diary and 3-month questionnaire) for analysis.
Replication	All attempts at replication were successful, through the use of Stata statistical software for data analysis by two investigators independently.
Randomization	All participants in this study received the BCG vaccine.<br>They had been randomised to receive the BCG vaccine in BRACE trial Stage 1. For the BRACE Trial, randomisation was done in a 1:1 ratio to receive or not receive BCG vaccination, using a web-based randomisation procedure on the Research Electronic Data Capture platform (REDCap),provided by an independent statistician.
Blinding	Data was analysed from participants who received the BCG vaccine, as the aim of the study was to determine the incidence, and the risk factors for the development, of BCG injection site abscess and regional lymphadenopathy. Hence investigators were not blinded to the vaccine group allocation.

Reporting for specific materials, systems and methods

We require information from authors about some types of materials, experimental systems and methods used in many studies. Here, indicate whether each material, system or method listed is relevant to your study. If you are not sure if a list item applies to your research, read the appropriate section before selecting a response.

Materials & experimental systems

n/a	Involved in the study	
☒	☐	Antibodies
☒	☐	Eukaryotic cell lines
☒	☐	Palaeontology and archaeology
☒	☐	Animals and other organisms
☐	☒	Human research participants
☒	☐	Clinical data
☒	☐	Dual use research of concern

Methods

n/a	Involved in the study	
☒	☐	ChIP-seq
☒	☐	Flow cytometry
☒	☐	MRI-based neuroimaging

Human research participants

Policy information about studies involving human research participants

Population characteristics	Participants were healthcare workers, ranging in age from 19 to 74 years old (median 41). The majority were female (76%).
Recruitment	This study was done using active surveillance data from a multicentre randomised controlled trial of BCG vaccination to reduce the impact of COVID-19 in healthcare workers (the BRACE trial; ClinicalTrials.gov NCT04327206; date of registration 31 March 2020). Healthcare workers (HCW) were recruited in Stage 1 of the BRACE trial in six hospitals in Australia from March to May 2020.<br>Procedures for recruitment into the BRACE Trial: participants received information about the BRACE trial via email, healthcare facilities notice board, website or social media. They evaluated their eligibility online, and accessed site-specific participant information and consent form prior to attending for enrolment. Eligibility was ascertained by research staff during the baseline visit where participants gave informed consent. Healthcare workers were eligible if working in healthcare settings during the COVID-19 pandemic or having face-to-face contact with patients. Exclusion criteria comprised any contra-indication to BCG, including previous significant local BCG adverse reaction.
Ethics oversight	Ethical approval was obtained from The Royal Children's Hospital Human Research Ethics Committee (HREC 62586).

Note that full information on the approval of the study protocol must also be provided in the manuscript.
